# Herpes Simplex Virus Type 1 Engages Toll Like Receptor 2 to Recruit Macrophages During Infection of Enteric Neurons

**DOI:** 10.3389/fmicb.2018.02148

**Published:** 2018-09-11

**Authors:** Paola Brun, Melania Scarpa, Chiara Marchiori, Jessica Conti, Andromachi Kotsafti, Andrea Porzionato, Raffaele De Caro, Marco Scarpa, Arianna Calistri, Ignazio Castagliuolo

**Affiliations:** ^1^Department of Molecular Medicine, University of Padua, Padua, Italy; ^2^Esophageal and Digestive Tract Surgery Unit, Veneto Institute of Oncology IOV–IRCCS, Padua, Italy; ^3^Department of Neurosciences, University of Padua, Padua, Italy

**Keywords:** neurotropic virus, neuromuscular dysfunction, gut dysmotility, inflammatory infiltrate, chemokines

## Abstract

*Herpes simplex* virus type 1 (HSV-1) is a widespread neurotropic pathogen responsible for a range of clinical manifestations. Inflammatory cell infiltrate is a common feature of HSV-1 infections and has been implicated in neurodegeneration. Therefore, viral recognition by innate immune receptors (i.e., TLR2) and the subsequent inflammatory response are now deemed key players in HSV-1 pathogenesis. In this study we infected with HSV-1 the enteric nervous system (ENS) of wild-type (WT) and TLR2 knock-out (TLR2^ko^) mice to investigate whether and how TLR2 participates in HSV-1 induced neuromuscular dysfunction. Our findings demonstrated viral specific transcripts suggestive of abortive replication in the ENS of both WT and TLR2^ko^ mice. Moreover, HSV-1 triggered TLR2-MyD88 depend signaling in myenteric neurons and induced structural and functional alterations of the ENS. Gastrointestinal dysmotility was, however, less pronounced in TLR2^ko^ as compared with WT mice. Interesting, HSV-1 caused up-regulation of monocyte chemoattractant protein-1 (CCL2) and recruitment of CD11b^+^ macrophages in the myenteric ganglia of WT but not TLR2^ko^ mice. At the opposite, the myenteric plexuses of TLR2^ko^ mice were surrounded by a dense infiltration of HSV-1 reactive CD3^+^CD8^+^INFγ^+^ lymphocytes. Indeed, depletion CD3^+^CD8^+^ cells by means of administration of anti-CD8 monoclonal antibody reduced neuromuscular dysfunction in TLR2^ko^ mice infected with HSV-1. During HSV-1 infection, the engagement of TLR2 mediates production of CCL2 in infected neurons and coordinates macrophage recruitment. Bearing in mind these observations, blockage of TLR2 signaling could provide novel therapeutic strategies to support protective and specific T-cell responses and to improve neuromuscular dysfunction in pathogen-mediated alterations of the ENS.

## Introduction

*Herpes simplex* virus type 1 (HSV-1) infections are endemic worldwide and seropositivity is deemed to exceed two thirds of the world’s population (Looker et al., 2015). In humans, HSV-1 is usually acquired during childhood by contact with contaminated mucosal secretions or skin lesions (Pellett and Roizman, 2013). After initial replication in epithelial cells, HSV-1 moves to cell body of sensory neurons by intra-axonal transport and establishes a lifelong latent infection primarily in trigeminal ganglia (Pellett and Roizman, 2013). However, studies demonstrated HSV-1 DNA in different neuronal structures innervating the gut (Rand et al., 1984; Gesser and Koo, 1997), suggesting more widespread HSV-1 dissemination than it is generally assumed. Latent HSV-1 infection is characterized by low level expression of viral antigens in infected neurons and by possible periodic reactivation leading to shedding of viral particles. Persistent HSV-1 infection is now supposed to drive chronic inflammatory responses that could cause the development of neuronal damage (Valyi-Nagy et al., 2000; Menendez et al., 2016). Although it is well accepted that innate and acquired immunity co-operate to restrain primary HSV-1 infection, to contain virus during latency, and to resolve spontaneous reactivation, the exact mechanism driving the development of inflammatory infiltrate and its consequence for the host are not completely clear.

Upon recognition of both viral nucleic acid and proteins, pattern recognition receptors of the innate immunity such as Toll-like receptors (TLRs) activate secretion of interferons, cytokines, and chemokines to shape inflammatory reactions and specific adaptive immune responses (Takeuchi and Akira, 2010; Iwasaki and Medzhitov, 2010; Thompson et al., 2011). The precise function of TLRs in the immune response to HSV-1 *in vivo* is still not fully understood since they appear to have a double-edged role in mediating both immune protection and immune pathology. Indeed, TLRs seem to either diminish or worsen virus-mediated disease depending on the pathogen, the cells involved, and the site of the infection. For instance, absence of TLR responses for deficiency in the TLR adaptor proteins MyD88 (myeloid differentiation primary response 88) or TRIF (TIR-domain-containing adapter-inducing interferon-β) results in encephalitis and host death following HSV-1 infection (Mansur et al., 2005; Sancho-Shimizu et al., 2011). TLR2 absence, however, reduces pathology and mortality generated by intraperitoneal or intranasal HSV-1 challenge (Kurt-Jones et al., 2004; Lima et al., 2010) whereas activation of TLR2 elicits protective responses in microglia cells (Aravalli et al., 2008). Moreover, TLR2 cooperates with TLR9 in controlling cytokine production both *in vitro* and in a mouse model of vaginal and intraperitoneal HSV-1 infection (Sørensen et al., 2008). Indeed, in the central nervous system TLR2 is essential for expression of inflammatory cytokines (i.e., TNF-α and IL-6) and chemokines by monocytes (i.e., CCL2), lymphocytes (i.e., CCL22 and CCL27) and neutrophils (i.e., RANTES) during viral infection (Aravalli et al., 2005; Zhou et al., 2008). Recruitment of macrophages and lymphocytes nearby infected neurons or glial cells represents a key step to limit replication and spread of neurotropic viruses. However, the inflammatory infiltrate potentially damages the neuronal tissue through direct or indirect mechanisms (Kodukula et al., 1999; White et al., 2016).

We recently reported that HSV-1 persistently infects the enteric nervous system (ENS) and engages a local immune response causing gut neuromuscular abnormalities (Brun et al., 2010; Brun et al., 2018). In this study, we tested the hypothesis that TLR2-dependent signals in enteric neurons orchestrate immune cells recruitment and mediate HSV-1 induced neuronal damage. Indeed, TLR2^ko^ mice failed to express CCL2 in HSV-1 infected enteric neurons. The missing macrophage recruitment allowed a strong T-cell response in the myenteric plexus of HSV-1 infected TLR2^ko^ mice and attenuated the virus-induced gastrointestinal dysfunction as compared with wild-type mice thus demonstrating the importance of neuronal TLR2 in coordinating the inflammatory response against HSV-1.

## Materials and Methods

### Viral Stocks Preparation

HSV-1 strain SC16 was propagated on Vero cells (ATCC^®^ CCL81^TM^, American Type Culture Collection, VA, United States), as previously described (Brun et al., 2018). Vero cells were maintained in Dulbecco’s Modified Eagle’s Medium (DMEM) supplemented with 10% heat-inactivated fetal bovine serum (FBS) and penicillin/streptomycin 1% (all from Gibco), at 5% CO_2_ and 37°C. HSV-1 stocks were prepared in DMEM 2% FBS and titrated on Vero cells by standard plaque technique. Viral stocks were adjusted to 1 × 10^8^ plaque-forming units (PFU)/mL.

### Animal Model of HSV-1 Infection and Mice Treatments

Wild-type (WT) C57BL/6J and TLR2 knockout (TLR2^ko^, B6.129-Tlr2tm1Kir/J) mice were obtained from Envigo Laboratories (Udine, Italy) and Charles River (Monza, Italy), respectively. Animals were kept at 22 ± 2°C with 12 hrs light/dark cycle and fed with standard rodent food and tap water. Eight weeks old mice received HSV-1 (1 × 10^2^ PFU) into one nostril. Animals were placed back in their cages and monitored daily for the occurrence of neurological abnormalities assessed using a validated scoring system (Garcia et al., 1995). Four weeks later, 1 × 10^7^ PFU of HSV-1 or equal volumes of Vero cell lysate (sham infection) were inoculated via intragastric (IG) route using a 24 gauge, 9-cm catheter. Mice were then sacrificed 1, 2, or 3 weeks following IG viral inoculum. Sham infected mice (control) were sacrificed at matching time points but since data were comparable, results were pooled and reported as one sham infected group. For CD8 depletion mice were intraperitoneally injected with 200 μg rat anti-mouse CD8 purified monoclonal antibody (clone 2.43) produced by hybridoma (ATCC^®^ TIB-210) and purified using Protein G PLUS-Agarose (Santa Cruz Biotechnology, Italy). The monoclonal antibody was administered 1 day after IG HSV-1 inoculation. As control mice received equal volumes of rat IgG. This study was carried out in accordance with the recommendations of National and European guidelines for handling and use of experimental animals. The protocol was agreed by the Animal Care and Use Committee of the Padova University under supervision of the Health Italian Ministry.

### Intestinal Whole Mount Preparation and Staining

For whole mount preparations, distal ileum was flashed with PBS, filled with 4% PFA and submerged in the same fixative for 1 h at 22°C (Brun et al., 2013). Then tissues were rinsed in PBS and one cm long specimens were dissected under microscope (Zeiss, Germany) to obtain the longitudinal muscle layer containing the myenteric plexus (LMMP). LMMP preparations were fixed on wax supports, washed in PBS containing 0.5% Triton-X100 and incubated in blocking buffer (2% bovine serum albumin, 0.5% Triton-X100 in PBS). Samples were stained with primary antibody (**Table 1**) at 4°C for 16 h and immune-complexes were detected using fluorescent labeled secondary antibodies (**Table 1**). Tissues were visualized using Leica TCSNT/SP2 confocal microscope.

**Table 1:** Primary and secondary antibodies used in the study.

Primary antibodies			
**Antigen (host)**	**Clone**	**Source**	**Application**
BetaIII-Tubulin (rabbit)	polyclonal	Couvance	IHC, FC, WM
Peripherin (rabbit)	polyclonal	Millipore	WM
S100β (rabbit)	EP1576Y	Millipore	WM
CCL2 (rat)	ECE.2	R&D systems	IHC, IF
β-Actin (mouse)	AC-15	Sigma-Aldrich	WB
CD3 (rat)	17A2	eBioscience	IHC, FC
CD4 (rabbit)	50134-R001	Sino Biological Inc	FC
CD8 (rabbit)	orb1269	Biorbyt Ltd	FC
IFNγ (rat)	XMG1.2	eBioscience	FC
F4/80 (rat)	CI:A3-1	Abeam	FC
CD11b (rabbit)	EPR1344	Abeam	IHC, FC
CD19 (rabbit)	C1C3	Gene Tex	FC
NK1.1 (rabbit)	PK136	Gene Tex	FC
TLR2 (mouse)	polyclonal	Abeam	WB, FC
TLR2 (rabbit)	polyclonal	Santa Cruz Biotech	IP
TLR2 (mouse)	T2.5	Abeam	IHC
MyD88 (mouse)	E-ll	Santa Cruz Biotech	WB
IL17 (rat)	eBiol7B7	eBioscience	FC

**Secondary antibodies**			

**Antigen (host)**		**Source**	**Application**

anti-rabbit (goat) PE		Chemicon	WM, FC, IHC
anti-rat (rabbit) FITC		Invitrogen	IHC, FC
anti-rabbit (goat) HRP		Sigma-Aldrich	WB, IHC
anti-mouse (goat) HRP		Sigma-Aldrich	WB
anti-rat (rabbit) HRP		Sigma-Aldrich	IHC
anti-rabbit (goat) APC		Chemicon	FC
anti-rat (rabbit) PE		Sigma-Aldrich	FC

*WB, western blot; IP, immunoprecipitation; WM, whole mount; IHC, immunohistochemistry; FC, flow cytometry.*

### Dissection of Longitudinal Muscle Myenteric Plexus

At the sacrifice, the abdomen was opened by a midline incision; the small intestine was exteriorized and aseptically removed. The explant was placed in oxygenated Krebs solution (126 mM NaCl, 25 mM NaHCO_3_, 2.5 mM KCl, 2.5 mM CaCl_2_, 1.2 mM MgCl_2_, 1.2 mM NaH2PO_4_, pH 7.2). Tissues were cut in pieces of ∼1 cm length. LMMP were peeled off and placed in ice-cold sterile Krebs solution. Samples were immediately snap-frozen in liquid nitrogen or subjected to enzymatic digestion to obtain single cells suspensions (Brun and Akbarali, 2018).

### Nucleic Acid Extraction and Analysis

Total RNA was extracted from LMMP (SV total RNA isolation system, Promega, Italy) and contaminating DNA was removed by digestion with DNase I (Promega). Quantitative PCR was performed using iTaq Universal SYBR Green One-Step Kit (Bio-Rad Laboratories, CA, United States) and the ABI Prism 7700 Sequence Detection System (PerkinElmer, Monza, Italy) with specific oligonucleotides (Universal Probe Library Assay Design Center, Roche Applied Science) listed in **Table 2**. Data were normalized to 18S ribosomal RNA (Rn18S) and results were represented as mean fold changes (Brun et al., 2010).

**Table 2:** Oligonucleotides and PCR conditions.

Oligonucleotide	sequence	Tm (°C)
LATs	Fw 5′-gacagcaaaacaataaggg-3′ Rv 5′-acgagggaaaacaataaggg-3′	60
ICP0	Fw 5′-ggtgtacctgatagtgggcg-3′ Rv 5′-gctgattgcccgtccagata-3′	60
ICP4	Fw 5′-atgacggggacgagtacgac-3′ Rv 5′-acgacgaggacgaagaggat-3′	56
VP16	Fw 5′-tgcgggagctaaaccacatt-3′ Rv 5′-tccaacttcgcccgaatcaa-3′	60
tk	Fw 5′-tagcccggccgtgtgaca-3′ Rv 5′-cataccggaacgcaccacacaa	60
gB	Fw 5′-ggctccttccgattctcc-3′ Rv 5′-ggtactcggtcaggttggtg-3′	60
gC	Fw 5′-ccaaacccaagaacaacacc-3′ Rv 5′-tgttcgtcaggacctcctct-3′	60
*Ccl2*	Fw 5′-gcctgctgttcacagttgc-3′ Rv 5′-caggtgagtggggcgtta-3′	60
*Cxcl11*	Fw 5′-cagctgctcaaggcttcctta-3′ Rv 5′-ctttgtcgcagccgttactc-3′	60
*Cxcl9*	Fw 5′-tcggacttcactccaacacag-3′ Rv 5′-agggttcctcgaactccacac-3′	60
*Rn18S*	Fw 5′-tcaagaacgaaagtcggagg-3′ Rv 5′-ggacatctaagggcatca-3′	60

*Fw, forward; Rv, reverse; Tm, melting temperature.*

### Gastrointestinal Transit

Fluorescein-isothiocyanate dextran solution (70,000 MW; 6.25 mg/mL in PBS; 100 μL/mice; MP Biomedicals LLC, CA, United States) was IG administered and mice were sacrificed after 60 min. Luminal contents were collected form the stomach, cecum, colon, and from 8 equal segments of the small intestine. Samples were clarified by centrifugation (10,000 ×*g*, 15 min, 4°C) and fluorescence was determined at 494/521 nm (Hitachi F2000; Hitachi, Tokyo, Japan). The percentage of FITC-dextran remaining in the stomach was calculated as the gastric emptying value. The geometric center of the fluorescent probe distributed along the ileum denotes the intestinal transit (Brun et al., 2013).

### Colonic Transit Measurement

Mice were slightly anesthetized with isoflurane (<1 min; Merial, France) and a single 2-mm glass bead was inserted into the distal colon at 2 cm from the anus (Brun et al., 2017). Colonic transit was assessed by monitoring the bead retention time.

### Histological Evaluation

Segments of ileum were placed in 10% buffered formalin for 24 h, embedded in paraffin and sectioned (5 μm thick). Sections were subjected to haematoxylin and eosin (H&E) staining and at least 10 fields per sample were examined using Leica microscope.

### Immunoblot Analysis

Layers containing the myenteric plexus were homogenized using a Retsch MM300 mixer in RIPA buffer added with protease inhibitors (Brun et al., 2013). Samples were incubated 30 min in ice and debris were removed by centrifugation (15.000 *× g*, 5 min at 4°C). Protein concentration was assessed in the supernatants using the bicinchoninic acid kit (Thermo Scientific, MA, United States). Protein extracts (1 mg) were incubated overnight at 4°C with anti-TLR2 antibody (**Table 1**) and immune-complexes were captured using Protein G–agarose beads (Santa Cruz Biotechnology, Italy). The precipitates were resolved on SDS–PAGE gel and blotted onto a PVDF membrane (Bio-Rad Laboratories). Membranes were incubated 1 h in 5% non-fat dry milk, 0.05% Tween20 in PBS and then probed with specific antibodies (**Table 1**). Immune-complexes were revealed by incubation with horseradish peroxidase (HRP)-conjugated secondary antibodies (**Table 1**) and enhanced chemiluminescent substrate (ECL, Millipore, Italy). Images were captured using Hyper Film MP (GE Healthcare, Italy). Control loading were obtained using antibody against mouse β-actin. Densitometric determination was performed using the ImageJ software (US National Institutes of Health).

### Immunohistochemistry

Paraffin embedded samples of ileum were cut, deparaffinized, and rehydrated (xylene 5 min; ethanol 100%, 95%, 70%, 1 min each) following standard procedures (Brun et al., 2010). To block endogenous peroxidase activity samples were exposed to 10% H_2_O_2_ and treated with citrate buffer (pH 9) for antigen retrieval as indicated. Tissue sections were incubated with universal blocking solution (Lab Vision Corporation, CA, United States) and proper antibody (**Table 1**; 1 h, 22°C) and immune-complexes were visualized using Dako Envision+ System-HRP labeled Polymer Detection (Dako, CA, United States) and 3,3′ diaminobenzidine tetrahydrochloride (DAB) chromogenic substrate. Sections were counterstained and observed. As negative control, we used either isotype-matched antibody of inappropriate specificity or we omitted the primary antibody.

### Immunofluorescence

After sacrifice, 10 cm long segments of the distal ileum were carefully removed, flashed with PBS and immediately placed in optimal cutting temperature mounting medium and frozen. Sections (5 μm) were obtained with a cryostat and thaw-mounted onto Superfrost Plus slides. Sections were air dried, fixed in 10% PFA for 10 min, washed twice in TBS, incubated for 30 min in blocking buffer, and then subjected to immunohistochemistry using a polyclonal antibody to TLR2 and βIII-tubulin (**Table 1**). Sections were extensively washed and then mounted with Prolong Antifade kit (Invitrogen) and imaged using a Leica TCSNT/SP2 confocal microscope (Leica Microsystems). Co-localization of immune-complexes was achieved by sequentially scanning the specimens with the individual lasers.

### Isolation of Mononuclear Cells and Enteric Neurons

For mononuclear cell isolation, LMMP were dissociated with collagenase type II from *Clostridium histolyticum* (10 mg/ml), dispase (62,5 μg/ml) and DNase I (10 μg/mL, all purchased from Sigma) for 10 min at 37°C (Brun et al., 2015; Brun and Akbarali, 2018). Tissue debris was filtered and cells were collected (900* × g* for 5 min), purified by density gradient using Ficoll-Hypaque (Sigma) and immediately stained for flow cytometry or cultured for 24 h at 37°C with or without UV-inactivated HSV-1 in the presence of GolgiPlug (DB Bioscience). For culture of enteric neurons, LMMP were dissociated in 1.3 mg/ml collagenase type II (Sigma) with 0.3 mg/ml bovine serum albumin (37°C, 15 min). Cell suspension was cultured on coverslips coated with laminin and poly-D-lysine (Sigma) in Neurobasal A media added with B-27 supplement, 1% FBS, 10 ng/mL nerve growth factor (BioLegend, Italy), penicillin/streptomycin 1% (Brun and Akbarali, 2018). On the seventh day of culture, neurons were washed, the culture medium replaced and incubated for 16 h with medium alone or containing UV-inactivated HSV-1. Then, cells were fixed in 4% PFA for 10 min and subjected to immunofluorescence staining for CCL2 and βIII-tubulin. Slides were imaged using a Leica TCSNT/SP2 confocal microscope (Leica Microsystems).

### Flow Cytometry Analysis and Intracellular Cytokine Staining

Freshly obtained macrophages and lymphocytes (10^6^/mL) were stained for 30 min in ice with proper antibodies (**Table 1**). In intracellular cytokine experiments, cells were then incubated in fixation and permeabilization buffer (eBioscience, Italy) containing the proper antibody (30 min, room temperature). Fluorescence was analyzed using BD FACSCanto^TM^ Flow Cytometry (BD Bioscience, Italy) and WinMDI 2.9 (Windows Multiple Document Interface for Flow Cytometry) program.

### CCL2 Quantification by ELISA

Layer containing the myenteric plexus were homogenized using a Retsch MM300 mixer in PBS (1:10 wt/vol) containing protease inhibitors (10 μg/mL aprotinin, 1 mmol/L phenylmethylsulfonyl fluoride, and 10 μg/mL leupeptin). Samples were centrifuged (10,000 ×*g*, 10 min at 4°C) and the supernatants were assessed for CCL2 protein using commercially available kit (eBioscience) and a microplate reader (Sunrise, Tecan; Switzerland).

### Statistical Analysis

Statistical analysis was performed using GraphPad Prism 3.03 software (GraphPad, San Diego, CA, United States). Data were reported as mean ± standard error of the mean (SEM) except for the fluorescent probe distribution in *in vivo* gastrointestinal transit experiments reported as median ± SEM. Statistical differences were assessed by one-way ANOVA and Bonferroni multicomparison *post hoc* tests. The statistical significance was reported in the legends of the figures. Statistical significance was considered for *P*-values of 0.05 or less.

## Results

### TLR2 Does Not Affect HSV-1 Replication in Murine LMMP

**FIGURE 1:**
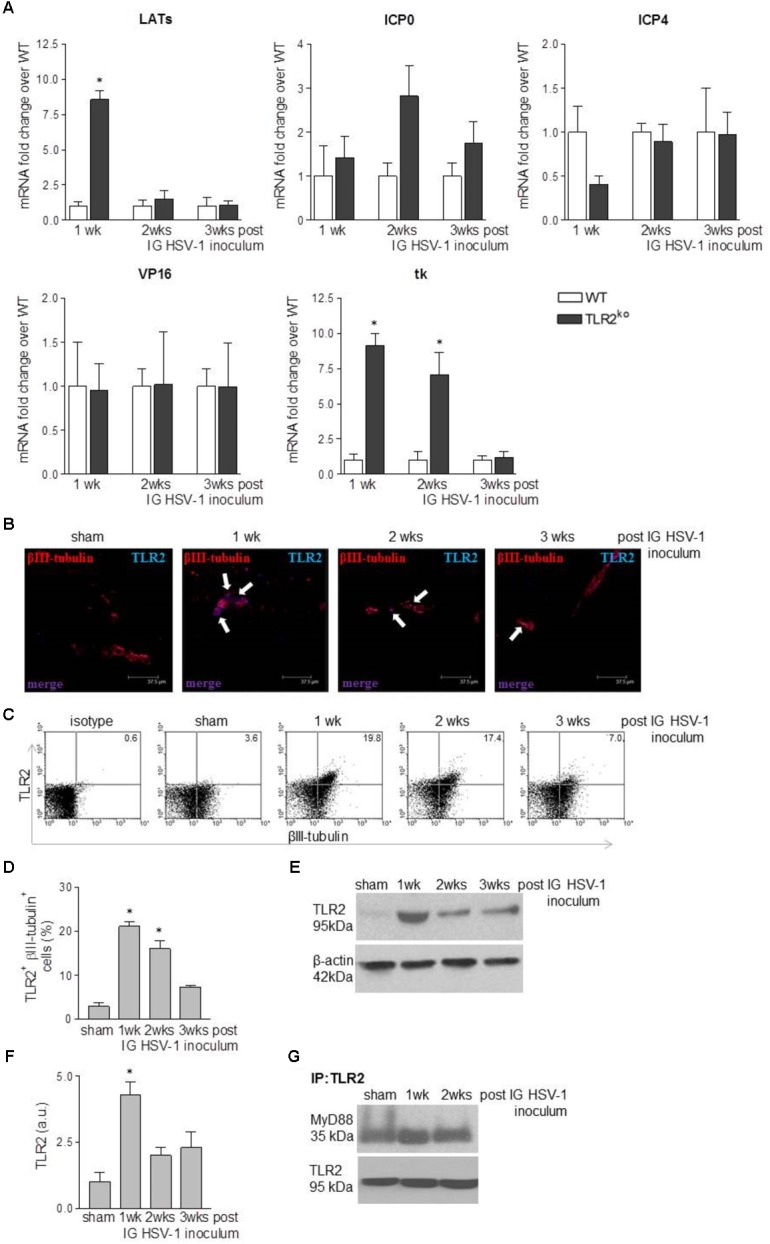
HSV-1 infection of the ENS triggers TLR2 activation. **(A)** One, two, and three weeks (wks) post IG inoculum of HSV-1, total RNA was purified from the LMMP of WT and TLR2^ko^ mice. Quantitative PCR was performed to evaluate the expression of HSV-1 latency-associated transcripts (LATs), infected cell protein (ICP0, ICP4), VP16, and thymidine kinase (tk) mRNA transcripts. Data were normalized to *Rn18S* and are reported as mRNA fold-change over WT mice. Sham: mice IG inoculated with Vero cell lysate. *n* = 6 mice per group. ^∗^ denotes *P* < 0.05 vs. WT mice at the same time of infection. **(B)** Dual-label immunohistochemistry showing expression of TLR2 and neural marker βIII-tubulin in frozen sections obtained from ileum of sham or HSV-1 infected WT mice. Scale bar: 37.5 μm.*Arrows indicate double positive stain. **(C)** Expression of TLR2 in βIII-tubulin^+^ cells isolated from LMMP of sham and HSV-1 infected WT mice. Cells were analyzed by flow cytometry and 10^4^ cells were collected. Representative dot plots are reported. **(D)** Mean percentage of TLR2 and βIII-tubulin positive cells acquired by flow cytometry as described in **(C)**. *n* = 6 mice per group. ^∗^ denotes *P* < 0.05 vs. sham infected mice. **(E)** Western blot analysis of TLR2 expression on protein extracts obtained from LMMP of sham and HSV-1 infected WT mice. β-actin was used as loading control. Representative images are reported. kDa = kilodalton. **(F)** Protein levels of TLR2 analyzed by western blot as described in **(E)** were determined by densitometry. Data were normalized to β-actin. *n* = 3 per group. ^∗^denotes *P* < 0.05 vs. sham infected mice. **(G)** Protein lysates from LMMP of sham and HSV-1 infected WT mice were immunoprecipitated with anti-TLR2 antibody. MyD88 expression was determined by Western blot. IP = immunoprecipitation.*

Several studies have reported the involvement of TLR2 in anti-HSV-1 response (Sørensen et al., 2008; Zolini et al., 2014) but no data are available about the role of TLR2 in controlling HSV-1 infection and replication in the ENS. We previously reported that HSV-1 retains infectivity following IG inoculum in WT mice (Brun et al., 2018). By comparing WT and TLR2^ko^ mice we found that HSV-1 infected the ENS of both animal strains (**Figure 1A**). Indeed, mRNA of HSV-1 latency-associated transcripts (LATs), ICP0, ICP4, VP16, and thymidine kinase (tk) were detectable in the LMMP of WT and TLR2^ko^ infected mice. Even if higher levels of virus mRNA transcripts (i.e., LATs and tk) were occasionally reported in TLR2^ko^ mice, expression of late genes (gB and gC) were undetectable in both murine strains (data not shown), suggesting abortive viral replication (see also Brun et al., 2010; Brun et al., 2018).

### Intragastric HSV-1 Inoculum Induces TLR2 Activation in LMMP

Since enteric neurons express functional TLR2 (Brun et al., 2013) and HSV-1 engages this receptor to initiate intracellular signaling (Leoni et al., 2012), in this study we at first asked whether TLR2 senses HSV-1 replication in the LMMP. Immunofluorescence analysis of ileum sections reported negligible expression of TLR2 in sham infected animals but TLR2 immunoreactivity increased in βIII-tubulin positive myenteric neurons at the first and second week post IG inoculum of HSV-1 (**Figure 1B**). Flow cytometry staining of cells dissociated from LMMP confirmed the increased expression of TLR2 in myenteric neurons of HSV-1 infected mice (**Figures 1C,D**). Finally, western blot analysis performed on extracts of LMMP preparations revealed a significant increased expression of TLR2 following IG inoculum of HSV-1 (**Figures 1E,F**). HSV-1 infection induced association and co-precipitation of TLR2 and the adaptor protein MyD88 (**Figure 1G**), demonstrating activation of functional TLR2-dependent signaling pathway in the LMMP of HSV-1 infected mice.

### TLR2^ko^ Mice Are Partially Protected Against HSV-1 Induced Gastrointestinal Dysfunction

**FIGURE 2:**
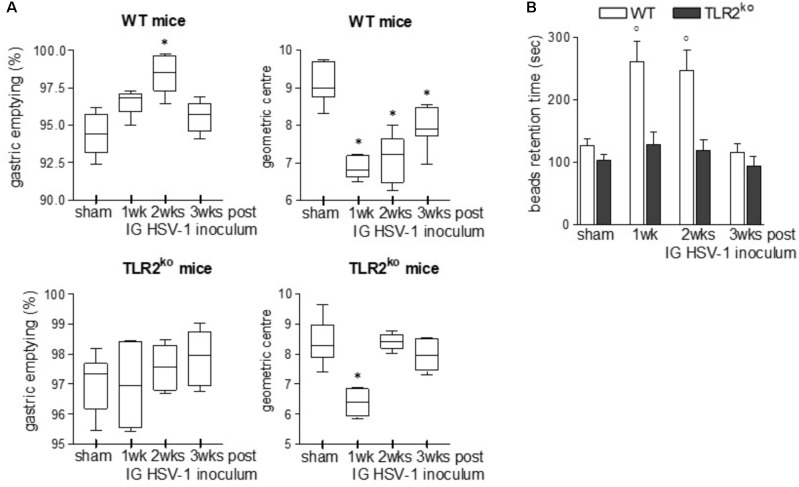
TLR2^ko^ mice experience mild gastrointestinal dysmotility following HSV-1 infection. **(A)** Sham and HSV-1 infected WT and TLR2^ko^ mice were IG injected with non-absorbable FITC-labeled dextran. Sixty minutes later mice were sacrificed. Gastric emptying was calculated as the percentage of probe retained into the stomach compared with the total amount of fluorescence in the gastrointestinal tract. Intestinal transit was reported as the geometric center of distribution of the fluorescent probe throughout the ileum. *n* = 8–10 mice per group. ^∗^denotes *P* < 0.05 vs. sham infected mice. **(B)** Time (seconds, sec) required for expulsion of a glass bead inserted at two centimeters from the anal verge. *n* = 8 mice per group. °denotes *P* < 0.02 vs. sham infected mice.

**FIGURE 3:**
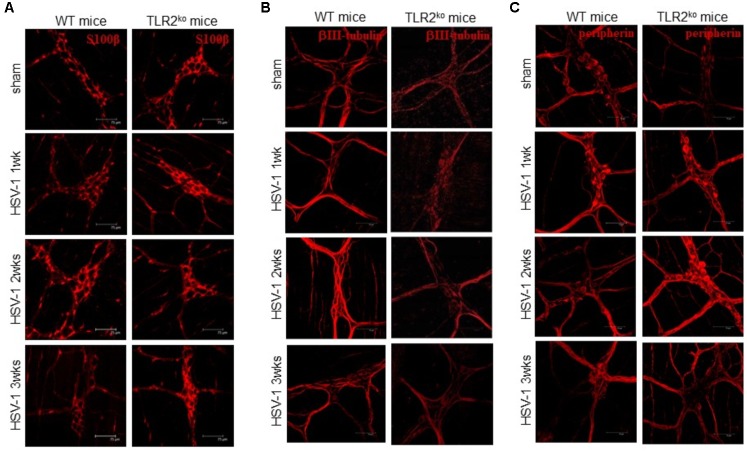
Alterations in myenteric plexus of TLR2^ko^ mice during HSV-1 infection. One, two, and three weeks post IG infection with HSV-1, samples of distal ileum was removed from WT and TLR2ko mice, fixed in neutral buffered formalin and processed to obtain whole mount preparations. Immunofluorescence analysis for **(A)** S100β (glial marker), **(B)** neurotubules βIII-tubulin, and **(C)** neurofilament peripherin was performed. Scale bars: 75 μm. Representative images of three separate experiments.

To examine the role of TLR2 in HSV-1 induced gastrointestinal dysfunction, we infected WT and TLR2^ko^ mice with HSV-1 and we monitored the animals for the onset, severity, and evolution of gastrointestinal neuromuscular dysfunction as well appearance of structural anomalies in the ENS. Following IG inoculum of HSV-1, WT mice reported quicker gastric emptying and persistent delay in intestinal transit whereas TLR2^ko^ mice had less pronounced alterations in gut motility (**Figure 2A**). At the first and second weeks post infection colonic motility, measured as the time required for bead expulsion, was slower in WT mice as compared with TLR2^ko^ mice (**Figure 2B**). However, in both WT and TLR2^ko^ mice infection with HSV-1 did not result in evidence of neurological or motor deficits (data not shown). However, we observed abnormalities in neuronal and glial cells of WT mice. Indeed, increased immunoreactivity of the glial marker S100β and enhanced expression of neuronal marker βIII-tubulin were observed 2 weeks post IG HSV-1 inoculum (**Figures 3A,B**). In contrast, S100β and βIII-tubulin immunoreactivity were not significantly modified by HSV-1 injection in TLR2^ko^ mice (**Figures 3A,B**). HSV-1 infection of the ENS did not significantly affect expression of the neuronal marker peripherin (**Figure 3C**). All together our data demonstrated that TLR2 activation contributes to functional and structural anomalies of the ENS during HSV-1 infection.

### TLR2 Is Mandatory for Macrophage Recruitment During HSV-1 Infection

**FIGURE 4:**
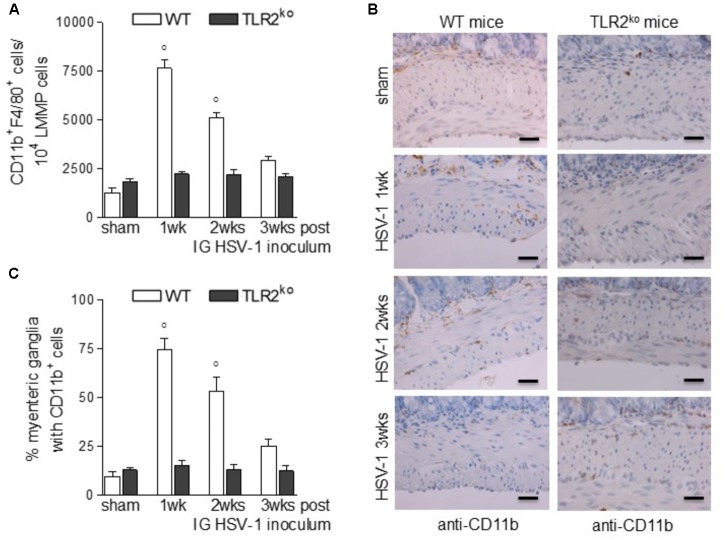
Lack of macrophage infiltration in HSV-1 infected TLR2^ko^ mice. **(A)** LMMP collected from sham and HSV-1 infected WT and TLR2^ko^ mice were enzymatically dissociated. The resulting cell suspensions were labeled with anti-CD11b and anti-F4/80 antibodies and analyzed by flow cytometry. 10^4^ events were collected. Data were graphed and reported as number of double positive cells. *n* = 8 mice per group. °denotes *P* < 0.02 vs. sham infected WT mice. **(B)** Immunohistochemistry for CD11b was performed on sections of distal ileum of WT and TLR2^ko^ mice. Representative images of three different experiments. Scale bars: 40 μm. **(C)** Myenteric plexuses showing contiguous or infiltrating CD11b^+^ cells were counted and normalized to the total number of myenteric plexuses. Data are reported as percentage. *n* = 6 mice per group. °denotes *P* < 0.02 vs. sham infected WT mice.

**FIGURE 5:**
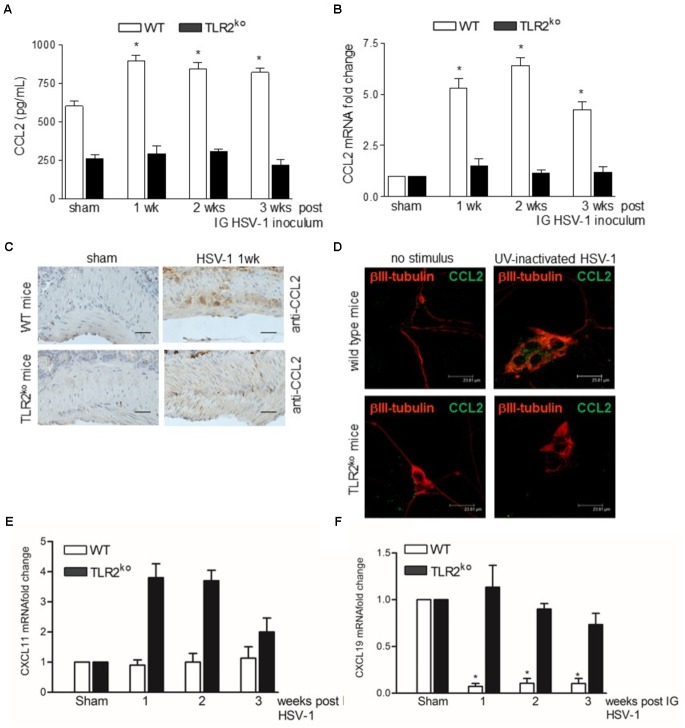
TLR2 is required for expression of CCL2 during HSV-1 infection. **(A)** LMMP collected from sham and HSV-1 infected WT and TLR2^ko^ mice were homogenized and CCL2 levels were quantified by ELISA. **(B)** Quantitative RT-PCR analysis of *Ccl2* mRNA transcripts. Data were normalized to *Rn18S* and reported as mRNA fold-change over sham infected mice. *n* = 6 mice per group. ^∗^denotes *P* < 0.05 vs. sham infected WT mice. **(C)** Immunohistochemistry for CCL2 on sections of ileum collected from sham and 1wk HSV-1 infected WT and TLR2^ko^ mice. Scale bars: 40 μm. **(D)** Immunofluorescence for CCL2 (green) and neuronal marker βIII-tubulin (red) on primary neurons isolated from LMMP of WT and TLR2^ko^ mice exposed to UV inactivated HSV-1 for 16 h. Scale bars: 23.8 μm. **(E)** Quantitative RT-PCR analysis of *Cxcl11* and **(F)** *Cxcl9* mRNA transcripts. Data were normalized to *Rn18S* and reported as mRNA fold-change over sham infected mice. *n* = 6 mice per group. ^∗^denotes *P* < 0.05 vs. sham infected WT mice.

Compelling evidence indicate that infiltrating macrophages play a relevant role in intestinal dysfunctions (Galeazzi et al., 2001; Kurt-Jones et al., 2004; Ellermann-Eriksen, 2005; Brun et al., 2018) since they regulate enteric neuronal activities (Muller et al., 2014). We therefore asked whether TLR2 is involved in macrophage recruitment in the LMMP of HSV-1 infected mice. Flow cytometry analysis of cells dissociated from LMMP and immunohistochemistry on sections of ileum revealed negligible macrophage recruitment nearby the myenteric ganglia of HSV-1 infected TLR2^ko^ mice whereas WT animals reported a significant increase in CD11b^+^F4/80^+^ macrophages contiguous to the myenteric ganglia (**Figures 4A–C**). Moreover, quantitative RT-PCR and ELISA performed in the LMMP of WT mice at 1–3 weeks post IG infection revealed increased mRNA and protein levels of CCL2, a chemoattractant factor involved in macrophage-driven tissue damage (Gosling et al., 1999; Huang et al., 2001; Brun et al., 2018). TLR2^ko^ mice completely failed to up-regulate CCL2 following HSV-1 exposure (**Figures 5A,B**). During HSV-1 infection, CCL2 positive cells were revealed in the myenteric ganglia of WT but not TLR2^ko^ mice (**Figure 5C**). Moreover in striking contrast to myenteric neurons cultured from WT mice, cells obtained from TLR2^ko^ mice completely failed to express CCL2 in response to HSV-1 challenge (**Figure 5D**). Overall these data indicate that TLR2 in enteric neurons is required to generate chemotactic signals for macrophages in response to HSV-1 infection. The lack of TLR2 signaling pathway skews the immune cells recruitment. Indeed, by quantitative RT-PCR performed on the LMMP of TLR2^ko^ mice we detected increased levels of *Cxcl11* and *Cxcl9*, chemokines involved in recruitment of T-cells (**Figures 5E,F**).

### TLR2^ko^ but Not WT Mice Develop Lymphocytic Infiltration in the Myenteric Ganglia Following HSV-1 IG Challenge

**FIGURE 6:**
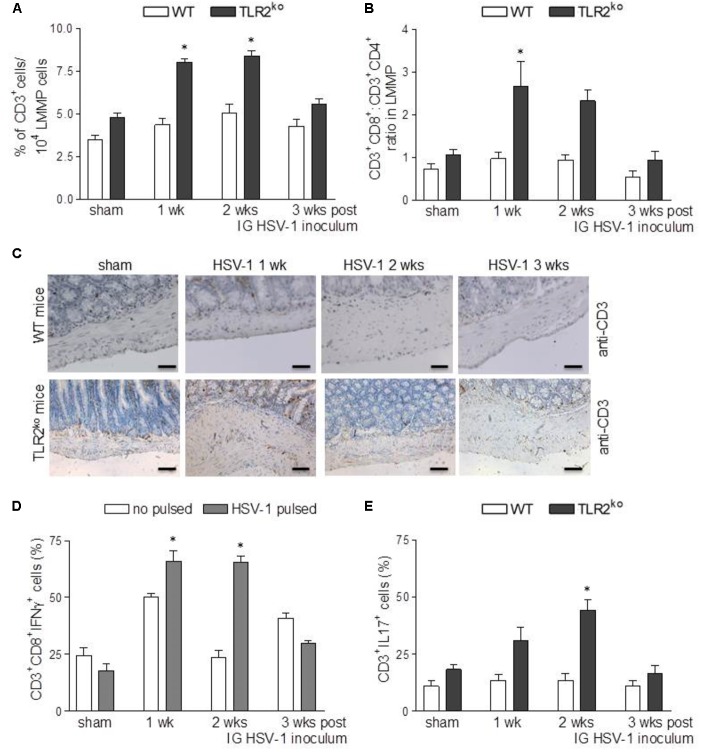
Strong lymphocyte recruitment in LMMP of TLR2^ko^ mice following HSV-1 exposure. **(A)** Freshly collected LMMP were enzymatically dissociated and the resulting cell suspensions were labeled with anti-CD3 antibody and analyzed by flow cytometry. CD3^+^ cells were expressed as percentage of 10^4^ collected events. *n* = 8 mice per group. ^∗^denotes *P* < 0.05 vs. sham infected TLR2^ko^ mice **(B)** Cell suspensions obtained from LMMP as described in **(A)** were labeled with anti-CD3, anti-CD4 or anti-CD8 antibodies and analyzed by flow cytometry. CD8:CD4 ratio of CD3^+^ cells was calculated. *n* = 8 mice per group. ^∗^denotes *P* < 0.05 vs. sham infected TLR2^ko^ mice **(C)** Sections of ileum obtained from sham and HSV-1 infected WT and TLR2^ko^ mice were subjected to immunohistochemistry for CD3. Scale bars: 40 μm. Representative images of three separate experiments. **(D)** Cell suspensions obtained from LMMP of TLR2^ko^ mice were cultured for 16 h in presence or absence of UV-inactivated HSV-1. Cells were then collected, labeled with anti-CD3, anti-CD8 and anti-IFNγ antibodies and analyzed by flow cytometry. 10^4^ events were collected in CD3^+^ gated cells. Data were graphed and reported as percentage of positive cells. *n* = 8 mice per group. ^∗^denotes *P* < 0.05 vs. no HSV-1 pulsed cells at the same time point. **(E)** Cell suspensions obtained from LMMP of sham and HSV-1 infected WT and TLR2^ko^ mice were labeled with anti-CD3 and anti-IL17 antibodies and analyzed by flow cytometry in 5 × 10^4^ events. *n* = 8 mice per group. ^∗^denotes *P* < 0.05 vs. sham infected TLR2^ko^ mice.

Along with other receptors of innate immunity, TLR2 has been reported to shape the adaptive immune response in different animal models of viral infection (Iwasaki and Medzhitov, 2010). Therefore, we next examined the recruitment of lymphocytes in HSV-1 infected myenteric plexus of WT and TLR2^ko^ mice. IG HSV-1 infection had no significant consequence in WT mice as regard the percentage and distribution of CD3^+^ cells and the CD8^+^:CD4^+^ ratio in the LMMP (**Figures 6A–C**). The percentage of CD3^+^/CD19^+^ cells and CD3^+^/NK1.1^+^ NK cells was comparable in HSV-1 infected mice and sham infected WT mice (data not shown). At the opposite, an abundant infiltrate of T cells was detected in the LMMP of TLR2^ko^ infected mice (**Figures 6A–C**), composed primary of HSV-1 responsive CD3^+^CD8^+^IFNγ^+^ cells (**Figure 6D**). Furthermore, following IG HSV-1 inoculum a significant increase in CD3^+^IL17^+^ infiltrated cells was detected in TLR2^ko^ mice as opposed to WT mice (**Figure 6E**).

### Lymphocytic Infiltration in the LMMP of TLR2^ko^ Mice Contributes to Neuromuscular Dysfunction Following HSV-1 IG Challenge

**FIGURE 7:**
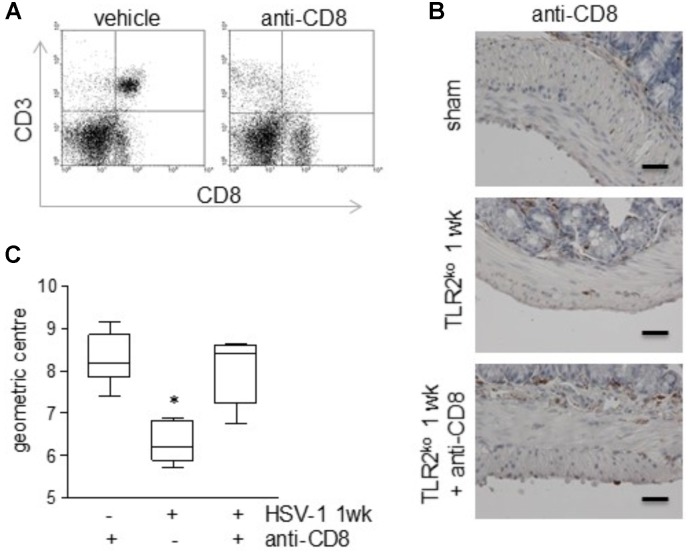
Depletion of CD8^+^ T cells in the LMMP of TLR2^ko^ mice ameliorates HSV-1-induced gut dysmotility. TLR2^ko^ mice were inoculated IG with HSV-1 and 1 day after received intraperitoneal injection of monoclonal anti-CD8 antibody. One week later, mice were sacrificed. **(A)** To confirm T cells depletion, cells isolated from LMMP were labeled with anti-CD3 and anti-CD8 antibodies and analyzed by flow cytometry. Representative dot plot. **(B)** Sections of ileum obtained from sham and 1 wk HSV-1 infected TLR2^ko^ mice were subjected to immunohistochemistry for CD3. Scale bars: 40 μm. Representative images of three separate experiments. **(C)** Distribution of FITC-labeled dextran was determined in the gut of TLR2^ko^ mice. Intestinal transit was reported as the geometric center of distribution of the fluorescent probe throughout the ileum. *n* = 6 mice per group. ^∗^denotes *P* < 0.05 vs. sham infected mice administered with anti-CD8 monoclonal antibody.

To elucidate the role of CD3^+^CD8^+^ cells on the mild gastrointestinal neuromuscular dysfunctions described in TLR2^ko^ mice during HSV-1 infection (**Figure 2**), infected animals were administered with monoclonal anti-CD8 antibody 7 day after IG HSV-1 injection and were sacrificed 1 week later. As reported in **Figures 7A,B**, administration of monoclonal anti-CD8 antibody was effective at depleting CD3^+^CD8^+^ cells in the LMMP. Depletion of CD8^+^ cells abolished HSV-1 induced gastrointestinal dysmotility as assessed by *in vivo* intestinal transit measurement (**Figure 7C**), suggesting that the CD3^+^CD8^+^ cells recruited in the LMMP account for the observed gastrointestinal alterations in TLR2^ko^ mice. Control mice administered with rat IgG did not reported significant intestinal alterations as compared with sham infected animals (data not shown).

## Discussion

Toll-like receptors are expressed by diverse cell population through the gut wall, including muscle cells, glial cells, enteric neurons, and immune cells (Brun et al., 2013). In the gut, the engagement of TLR2 by PAMPs or endogenous ligands regulates tissue development, homeostasis, and repair but also triggers inflammatory responses against a wide spectrum of microorganisms (Cario et al., 2007). In this study we demonstrated that during HSV-1 infection of the ENS, TLR2 expressed on enteric neurons orchestrates the recruitment of T lymphocytes involved in neuropathology and gut motor dysfunctions (**Figures 2**, **3**).

TLR2 is strategic in switching on the inflammatory response to HSV-1 infection (Kurt-Jones et al., 2004; Sørensen et al., 2008). Thus, *in vitro* HSV glycoproteins gH/gL and gB engages TLR2 on epithelial cells and neurons to elicit intracellular NF-κB signaling (Leoni et al., 2012). Moreover, expression of TLR2 is enhanced in the hindbrain of mice infected with HSV-2 (Boivin et al., 2002) whereas TLR2 activation during acute HSV-1 encephalitis in neonatal and adult mice significantly boost the inflammatory damage in the nervous tissue (Kurt-Jones et al., 2004; Aravalli et al., 2005). Here we reported that in WT mice HSV-1 infection of the ENS induced TLR2 upregulation on myenteric neurons and TLR2/MyD88 dependent activation in the LMMP (**Figure 1**) leading to production of CCL2 and macrophage recruitment (**Figures 4**, **5**). Indeed, in TLR2^ko^ mice infection of the ENS with HSV-1 induced a skewed chemokine response compared with WT mice (**Figure 5**) suggesting that HSV-1 recognition through TLR2 plays a pivotal role in coordinating the initial anti-viral inflammatory response.

Expression by embryonic and adult neurons of different functional Pattern Recognition Receptors (Barajon et al., 2009; Brun et al., 2013, 2015) enables detection of microbial signals (i.e., bacterial PAMPs or viral components) and allows secretion of specific soluble factors to generate appropriate protective microenvironments (Burgueño et al., 2016). In the ENS, enteric neurons express TLRs and play a primary role in shaping gastrointestinal inflammatory responses since they directly integrate a variety of environmental signals including bacterial toxins or microbial PAMPs (Pothoulakis et al., 1998; Burgueño et al., 2016). Indeed, enteric neurons directly recruit inflammatory cells through the production of cytokines, growth factors, and chemokines (Burgueño et al., 2016; Gabanyi et al., 2016). In neurons, lack of TLR3 empowers protective responses to neurotropic viruses such as West Nile virus and Japanese encephalitis virus (Daffis et al., 2008; Fadnis et al., 2013) whereas TLR2 elicits innate responses following HSV exposure (Gianni et al., 2013). In the present study we demonstrated that at early time of infection, TLR2-mediated HSV-1 recognition induced enteric neurons to produce CCL2 and drove a robust macrophage recruitment from the bloodstream (**Figures 4**, **5**; Chen et al., 2003; Dessing et al., 2007). Indeed, neurons of TLR2^ko^ mice failed to express CCL2 in response to HSV-1 thus reducing macrophage infiltration (Gosling et al., 1999; Muessel et al., 2002; Kurt-Jones et al., 2004). Therefore, through TLR2 signaling HSV-1 appears to favor macrophage recruitment and to minimize lymphocytes activation which instead prevails in the myenteric plexus of TLR2^ko^ mice (**Figure 6**). Consistent with our results, we suggested that CCL2 has a dominant role in the neuronal-mediated response to HSV-1 infection (Brun et al., 2018). Indeed, during viral encephalitis neurons secrete CXCL10 to recruit anti-viral effector CD8^+^ T cells, demonstrating that neuronal cells specifically shape immune responses against invading pathogens (Patterson et al., 2003; Klein et al., 2005). Similarly, we found that in absence of a macrophage-mediated immune response the recruitment of CD8^+^IL17^+^ lymphocytes increased in TLR2^ko^ mice compared with WT animals (**Figure 6**).

Upon recognition of viral antigens carried by MHC class I on antigen presenting cells (APCs), naïve CD8^+^ T cells differentiate into Tc1, Tc2, or Tc17 cells and express high levels of KRLG-1 (killer lectin-like receptor subfamily G member 1) and the pro-inflammatory cytokine IFN-γ that mediate the biological effects (Bettelli et al., 2008). Tc17 cells have a pivotal role in the controlling of infection diseases (Kolls and Lindén, 2004) and have been involved in experimental autoimmune encephalomyelitis (Komiyama et al., 2006; Nichols et al., 2009). An earlier report described IL-17 expression in the central nervous system of mice chronically infected with *Toxoplasma gondii* (Stumhofer et al., 2006) whereas IL-17 producing cells have been associated to HSV-1 uveitis (Yu et al., 2013). Alike our findings in the ENS, IL-17 significantly increased in abscesses of the central nervous system in TLR2^ko^ mice (Kielian et al., 2005; Stenzel et al., 2008; Nichols et al., 2009), suggesting that the Tc17 infiltrate compensates the loss of TLR2-dependent signals in controlling infections. Indeed, the increased presence of Tc17 cells in the myenteric plexus of HSV-1 infected TLR2^ko^ mice could be the result of a different milieu of soluble factors shaped by the lack of TLR2 signaling. Thus, TLR2-mediated signals have been reported to affect transcription factors that promote/repress Tc17 development (Ivanov et al., 2006; Moisan et al., 2007) and expression of soluble factors (i.e., IL-27) reducing Th17 development (Stumhofer et al., 2006; Batten et al., 2006). In this study we found that the absence of TLR2 diminishes the recruitment of macrophages (**Figure 4**), APCs also involved in production of Tc17 regulators (Mangan et al., 2006; Dong, 2008).

As major finding, in this work we confirmed the harmful effect of TLR2 responses in enteric neurons during microbial insults. Indeed, we provided original evidence that TLR2 signaling on neuronal cells plays an important role in HSV-1 induced neuropathogenesis in the ENS since TLR2 activation shapes the inflammatory infiltrate through a precise chemokine milieu and supports macrophage recruitment which results in a more severe neuronal damage.

## Author Contributions

PB and IC designed the research, performed the research, analyzed the data, and wrote the manuscript. MS, CM, JC, and AK performed the research. AP, RDC, MS, and AC critically revised the manuscript. All authors listed approved the manuscript for publication.

## Conflict of Interest Statement

The authors declare that the research was conducted in the absence of any commercial or financial relationships that could be construed as a potential conflict of interest.
